# Advanced-stage cervix cancer: rapid tumour growth rather than late diagnosis

**DOI:** 10.1054/bjoc.2000.1336

**Published:** 2000-08-16

**Authors:** P Symonds, B Bolger, D Hole, J H Mao, T Cooke

**Affiliations:** Beatson Oncology Centre, University Department of Oncology, Leicester Royal Infirmary, Leicester; University Department of Surgery, Glasgow Royal Infirmary, Glasgow; West of Scotland Cancer Surveillance Unit, Glasgow; Beatson Institute for Cancer Research, Bearsden, Glasgow, UK

**Keywords:** cervical cancer, diagnosis, proliferation rates

## Abstract

Either diagnostic delay or tumour biology are possible factors governing the degree of spread at diagnosis of cervical cancer. To try to identify the most important parameter contributing to advanced stage, the duration of symptoms were recorded from patients scheduled for radiotherapy (*n* = 141) or radical hysterectomy (*n* = 36). In 146 cases tumour proliferation rates were evaluated following in vivo labelling with the DNA precursor BrdUrd. For symptomatic patients there was no association between duration of symptoms and stage at presentation. There was a significant trend for patients with increasing tumour stage to have more rapidly proliferating tumours with higher mean labelling index (LI) measurements (*P* = 0.001) and a shorter mean potential doubling time (Tpot) (*P* = 0.023). Socio economic deprivation may be associated with shorter Tpot values. The conclusion from this data is that stage at diagnosis is more dependent on the biological behaviour of the tumour, as expressed by proliferation rates, than delay in presentation. © 2000 Cancer Research Campaign


*British Journal of Cancer* (2000) **83**(5), 566–568
© 2000 Cancer Research Campaign
doi: 10.1054/ bjoc.2000.1336, available online at http://www.idealibrary.com on IDEAL®

### Advanced-stage cervix cancer: rapid tumour growth rather than late diagnosis

**P Symonds[1], B Bolger[2], D Hole[3], JH Mao[4] and T Cooke[2]**

[1]Beatson Oncology Centre, University Department of Oncology, Leicester Royal Infirmary, Leicester; [2]University Department of Surgery, Glasgow Royal Infirmary, Glasgow; [3]West of Scotland Cancer Surveillance Unit, Glasgow; [4]Beatson Institute for Cancer Research, Bearsden, Glasgow, UK

**Summary** Either diagnostic delay or tumour biology are possible factors governing the degree of spread at diagnosis of cervical cancer. To try to identify the most important parameter contributing to advanced stage, the duration of symptoms were recorded from patients scheduled for radiotherapy ($n$ = 141) or radical hysterectomy ($n$ = 36). In 146 cases tumour proliferation rates were evaluated following in vivo labelling with the DNA precursor BrdUrd. For symptomatic patients there was no association between duration of symptoms and stage at presentation. There was a significant trend for patients with increasing tumour stage to have more rapidly proliferating tumours with higher mean labelling index (LI) measurements ($P$ = 0.001) and a shorter mean potential doubling time (Tpot) ($P$ = 0.023). Socio economic deprivation may be associated with shorter Tpot values. The conclusion from this data is that stage at diagnosis is more dependent on the biological behaviour of the tumour, as expressed by proliferation rates, than delay in presentation. © 2000 Cancer Research Campaign

**Keywords**: cervical cancer; diagnosis; proliferation rates

It is often presumed that cancers found to be late-stage at diagnosis are due to late-stage at presentation by the patient or diagnostic delay. Rapid tumour growth is an alternative explanation but it is difficult to measure the rate of growth of tumours. Gross volume doubling time estimates can only be made accurately in some situations, such as using serial chest radiographic measurements, and this is only possible if the patient is not receiving anti-cancer treatment. Tumour volume doubling time is a balance between tumour growth and cell loss due to apoptosis, necrosis and sloughing. There is no accurate way of measuring cell loss factor. However, it is possible to measure the cell birth rate in vivo by labelling tumours with DNA precursor bromodeoxyuridine (BrdUrd). Flowcytometry is used to measure the proliferative parameters BrdUrd labelling index (LI) DNA synthesis time (Ts) and potential doubling time (Tpot). Tpot is defined as the time in which the cell population would double if there was no cell loss. Previously we have shown that labelling index measured during this non-toxic assay has been shown to be an independent prognostic variable in a multi-variate analysis and a predictor of local control following radical radiotherapy (Bolger et al, 1996). Other investigators have reported similar observations (Tsang et al, 1999).

In this study we have tried to answer whether diagnostic delay or rapid tumour proliferation is responsible for late-stage cervical cancer.

### MATERIALS AND METHODS

Over a 20-month period all patients in the west of Scotland with cervical cancer referred for either radiotherapy (Beatson Oncology Centre, $n$ = 141) or radical surgery (Stobhill Hospital or Glasgow Royal Infirmary, $n$ = 36) were interviewed before treatment. The duration of symptoms (irregular vaginal bleeding, discharge or pain) and date of last normal cervical smear were recorded. Socioeconomic status was derived from the residential postal code (Carstairs and Morris, 1991). The tumour stage was assessed according to FIGO criteria during an examination under anaesthetic. Written informed consent was obtained from 146 patients for administration of 200 mg of bromodeoxyuridine (BrdUrd) 6–8 h before the staging EUA (approved by Ethics Committee). A mean of 2.8 biopsies (range 1–6) were obtained from each tumour and fixed in 70% ethanol. A 50 mg portion of each biopsy was disaggregated by pepsin to produce a nuclear suspension and the incorporated BrdUrd was revealed by partial denaturation of the DNA by hydrochloric acid. The BrdUrd was detected using a mouse anti-BrdUrd monoclonal antibody (Dako) and a FITC conjugated goat anti-mouse antibody (Sigma Chemicals). The DNA was fluorescently stained with propidium iodide and samples were analysed on a Coulter Epics Profile II flow cytometer for S-phase duration (Ts). BrdUrd labelling index (LI) and tumour potential doubling time (Tpot) were calculated (Bolger et al, 1993). Both rate of growth parameters were skewed, with longer tails to the right. A logarithmic transformation brought each distribution sufficiently close to normality and an analysis of variance was undertaken to test for a linear trend across the four stages.

### RESULTS

Prior to diagnosis, 53% (95) of patients had never had a cervical smear. Such patients tended to be older (mean age 63.3 years). Only 27.6% (49) had a smear taken within the previous 5 years. However, it is noteworthy that the diagnosis of asymptomatic invasive cancer was made following a routine smear in 13 patients (5.7%) (Table 1). We could find no association with deprivation

*Received 25 March 1999*
*Revised 6 April 2000*
*Accepted 17 May 2000*

*Correspondence to:* RP Symonds

**Table 1** Cervical smear history

| Last smear | Number of women | Mean age (years) |
|---|---|---|
| No previous smear | 95 | 63.3 |
| > 5 years ago | 33 | 51.7 |
| 3–5 years ago | 29 | 46.1 |
| <3 years ago | 20 | 41.2 |

**Table 2** Deprivation category in relation to compliance to cervical screening programme. Information only available for 163 patients, no postal code for four patients, deprivation category not determined for 10 patients (sparsely populated areas)

| Smear history | Deprivation category | | |
|---|---|---|---|
| | Affluent | Average | Deprived |
| ≤ 5 years (*n* = 43) | 29.7% | 31.3% | 19.4% |
| > 5 years (*n* = 120) | 70.3% | 68.7% | 80.6% |

$\chi^2$ = 2.6, *P* = 0.28

**Table 3** Duration of symptoms in relation to tumour stage

| Duration of symptoms | Stage | | Total |
|---|---|---|---|
| | Early (I or IIa) (*n* = 45) | Late (≥IIb) (*n* = 99) | |
| No symptoms | 17.8% (8) | 5.1% (5) | 13 |
| ≤3 months | 37.8% (17) | 50.5% (50) | 67 |
| 3–12 months | 28.9% (13) | 28.3% (28) | 41 |
| > 1 year | 15.5% (7) | 16.1% (16) | 23 |

All cases symptomatic vs asymptomatic $\chi^2$ = 4.65, *P* = 0.03; Symptomatic cases only test for trend $\chi^2$ = 0.38, *P* = 0.54

**Table 4** Duration of symptoms in relation to deprivation category

| Duration of symptoms | Deprivation category | | |
|---|---|---|---|
| | Affluent (*n* = 37) | Average (*n* = 64) | Deprived (*n* = 62) |
| No symptoms | 16.2% (6) | 18.7% (12) | 22.5% (14) |
| ≤ 3 months | 56.8% (21) | 34.4% (22) | 38.7% (24) |
| 3–12 months | 27.0% (10) | 29.7% (19) | 19.4% (12) |
| > 1 year | 0 | 17.2% (11) | 19.4% (12) |

$\chi^2$ = 11.8, *P* = 0.066

category and attendance for cervical screening (Table 2). A highly significant difference in the stage distribution exists when comparing symptomatic and asymptomatic cases (*P* = 0.03, Table 3). However, amongst the symptomatic cases 94/131 (72%) were late-stage and this group showed no evidence of a trend between stage and duration of symptoms (*P* = 0.54).

There was a trend suggesting women in higher deprivation categories had a long duration of symptoms but this was not statistically significant (Table 4). There was no correlation between stage at diagnosis and deprivation (*P* = 0.34).

Crude and adjusted labelling index (LI corrected for ploidy) was calculated either as the average LI from multiple biopsies or as a maximum value from one biopsy. The average LI may be less than the maximum recorded result owing to normal cell contamination and necrotic or slowly dividing tumour cells in the biopsies assayed. The maximum labelling index is representative of the most rapidly dividing part of the tumour which is probably the tissue most likely to decide the ultimate clinical outcome. There is a statistically significant trend for patients with increasing tumour stage to have higher mean LI measurements both for average values (*P* = 0.035), and for maximum measurements (*P* = 0.001, Figure 1). The mean potential doubling times show the same trend with the mean Tpot decreasing with increasing stage. This relationship is not significant for average values (*P* = 0.10) but is significant when maximum values only are considered (*P* = 0.023, Figure 1). The stronger relationship with stage is observed with LI, and when both LI and Tpot are included in a multivariate model only LI is independently associated with stage.

Data looking at the role of socioeconomic deprivation is contradictory. The Tpot values for socioeconomically deprived patients are significantly shorter than those for affluent patients when average Tpot values from all biopsies are considered (*P* = 0.04), but the relationship was far from significant for maximum values.

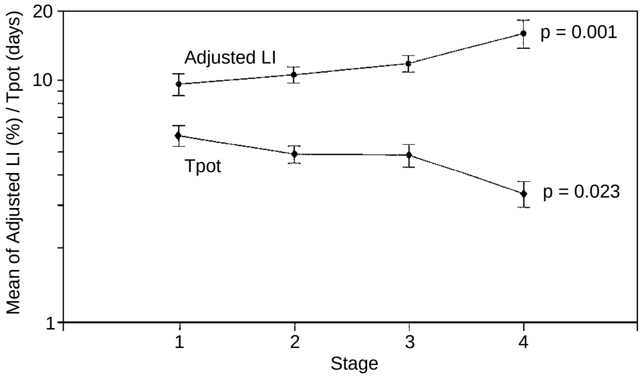

**Figure 1** Relationship between stage, maximum adjusted labelling index and Tpot (± SE)

### DISCUSSION

We know of no other studies comparing tumour stage, diagnostic delay and a reliable measurement of tumour proliferation. Our study was carried out during a time-period when a considerable effort was made in the west of Scotland to improve screening for cervical cancer. If the asymptomatic cases detected by screening are excluded, there is no relationship between duration of symptoms and the stage of detection. However, we do observe a statistically significant trend for advanced tumours to have higher labelling indices. There is also a trend for advanced tumours to have shorter potential doubling times if maximum LI measurements are used to calculate Tpot. This suggests that advanced-stage tumours have more rapid proliferation rates, shorter potential doubling times and by inference more rapid growth. Tumour stage at diagnosis seems more influenced by the biology of the cancer rather than diagnostic delay.

Duration of vaginal bleeding has been shown to be associated with tumour stage in endometrial cancer (Obermair et al, 1996). However, no association was found between delay and tumour stage at diagnosis in Belgian patients suffering from squamous cancer of the head and neck (Dhooge et al, 1996), a group of

© 2000 Cancer Research Campaign tumours with some biological features in common with cervical cancer including a rapid potential doubling time (Wilson et al, 1995). The incidence of cervical cancer in the most socioeconomically deprived is three times more common than in the most affluent in the west of Scotland. Moreover, the more deprived are more likely to die, as the cancer tends to be more advanced at diagnosis (Lamont et al, 1993). Poor survival of cervical cancer patients in New York City also seemed to be associated with low income (Serur et al, 1995).

Our data suggests that two factors may account for the poor prognosis of deprived patients. There is a non-significant trend for more deprived patients to have a greater duration of symptoms. However, proliferation rates may be more rapid in less affluent women, but this observation requires further confirmation in a larger group of patients.

The increased incidence and possibly a more malignant phenotype in the less affluent may be accounted for by lifestyle factors including smoking and diet. Low serum vitamin A has been shown to be a risk-factor in the development of cervical cancer (Eckhert et al, 1995). Interestingly, smoking has been associated with low levels of plasma beta-carotene. An additional finding was that, in contrast to non-smokers, dietary beta-carotene supplements failed to increase plasma levels in smokers (Palan et al, 1998). There is a very strong association between human papilloma virus infection and cervical cancer (Lombard et al, 1998). Retinoids are important regulators of human papilloma virus and can inhibit growth of cervical carcinoma cells in culture by transcription repression (Bartsch et al, 1992).

Recently the Office of National Statistics has shown (Coleman et al, 1999) that in England and Wales the affluent had a better survival after cancer treatment in 44 out of 47 cancers studies. Clearly this effect may be multifactorial, including possible better access to medical care by the affluent, but lifestyle factors shaping the tumour phenotype may also be important.

### ACKNOWLEDGEMENTS

This study was funded by the Scottish Home and Health Department. We would like to thank Drs Davis, Kennedy, Habeshaw and Reed for allowing patients to be recruited into this study and obtaining tumour biopsies.

### REFERENCES

Bartsch D, Boye B, Baust Çzur Hausen H and Schwarz E (1992) Retinoic acid-mediated repression of human papilloma virus 18 transcription and different ligand regulation of the retinoic acid receptor beta gene in non tumorigenic and tumorigenic Hela hybrid cells. *EMBO J* **22**: 83–91

Bolger BS, Cooke TG, Symonds RP, MacLean AB and Stanton PD (1993) Measurement of cell kinetics in cervical tumours using bromodeoxyuridine. *Br J Cancer* **68**: 166–171

Bolger BS, Symonds RP, MacLean AB, Stanton PD, Cooke TG, Burnett P and Kelly P (1996) Prediction of radiotherapy response of cervical carcinoma through measurement of proliferation rate. *Br J Cancer* **74**: 1223–1226

Carstairs V and Morris R (1991) *Deprivation and Health in Scotland*. Aberdeen University Press: Aberdeen

Coleman MP, Babb P, Damiecki P, Grosclaude P, Honjo S, Jones J, Gerhjardt K, Picard A, Quinn M, Sloggett A and De Stavola B (1999) *Cancer Survival trends in England and Wales: Deprivation and NHS Region Office for National Statistics*. pp. 88–100 HMSO: London

Dhooge IJ, Albers FWJ and van Cauwenberge PB (1996) Clinical characteristics and diagnostic delay of head and neck cancer: results from a prospective study in Belgium. *Eur J Surg Oncol* **23**: 354–358

Eckhert RL, Agrawal C, Hembree JR, Choo CK, Sizemore N, van Leyen SA and Rorke EA (1995) Human Cervical Cancer: Retinoids, interferon and human papilloma virus. *Adv Exp Med Biol* **375**: 31–44

Lamont DW, Symonds RP, Brodie MM, Nwabineli and Gillis CR (1993) Age, socio-economic status and survival from cancer of cervix in the west of Scotland 1980–1987. *Br J Cancer* **67**: 351–357

Lombard 1 Soloman AV, Validire P, Zafrandi B, de la Rochefordiere A, Clough K, Farre M, Pouillart P and Sastre-Garau X (1998) Human papilloma virus genotype as a major determinant of the course of cervical cancer. *J Clin Oncol* **16**: 2613–2619

Obermair A, Hanzal E, Schreiner-Frech I, Buxbaum P, Bancher-Todesca D, Thoma M, Kurz C, Vavra N, Gitsch G and Svelda P (1996) Influence of delay on established prognostic factors in endometrial cancer. *Anticancer Res* **16**: 947–950

Palan PR, Chang CJ, Mikhail MS, Ho GYF, Basu J and Romney SL (1998) Plasma concentrations of micronutrients during a nine month clinical trial of beta carotene in women with precursor cervical cancer lesions. *Nutr Cancer* **301**: 46–52

Serur E, Fruchter RG, Maiman M, McGuire J, Arrastia CD and Gibbon MD (1995) Age, substance abuse and survival of patients with cervical carcinoma. *Cancer* **75**: 2530–2538

Tsang RW, Wong CS, Fyles AW, Levin W, Manchal LA, Milosevic M, Chapman W Li Y and Pintilie M (1999) Tumour proliferation and apoptosis in human cervix carcinoma II: correlation with clinical outcome. *Radiother Oncol* **50**: 93–101

Wilson GD, Dische S and Saunders MI (1995) Studies with bromodeoxyuridine in head and neck cancer and accelerated radiotherapy. *Radiother Oncol* **36**: 189–197

© 2000 Cancer Research Campaign
